# A phase I/II clinical trial of ex-vivo expanded human bone marrow derived allogeneic mesenchymal stromal cells in adult patients with perianal fistulizing Crohn’s Disease

**DOI:** 10.1186/s13287-024-03746-9

**Published:** 2024-05-14

**Authors:** Shekhar Swaroop, Sudheer Kumar Vuyyuru, Bhaskar Kante, Peeyush Kumar, Sandeep Kumar Mundhra, Umang Arora, Ankur Goyal, Devasenathipathy Kandasamy, Raju Sharma, Kavirajan Kabilan, Saurabh Kedia, Nihar Ranjan Dash, Vineet Ahuja

**Affiliations:** 1 Department of Gastroenterology, AIIMS, New Delhi, India; 2 Department of Medical Gastroenterology, KIMS Hospitals, Hyderabad, India; 3 Department of Radiodiagnosis and Interventional Radiology, AIIMS, New Delhi, India; 4 Department of Gastrointestinal Surgery, AIIMS, New Delhi, India

**Keywords:** Perianal CD, Fistula, Stem cell

## Abstract

**Background:**

Perianal fistulas (PF) affect one-third patients with Crohn’s disease (CD) with limited therapeutic options. There is dearth of literature on safety and efficacy of bone marrow-derived mesenchymal stromal cells (BMSCs) in this population.

**Methods:**

An open-label, phase I/II, single-arm study was conducted involving local administration of human allogeneic bone marrow-derived mesenchymal stromal cells in perianal fistula of patients with Crohn’s disease refractory to standard therapies. Clinical severity and biomarkers were assessed at baseline and periodically until week 104 , and MRI at week 24 and 104. Primary and secondary objectives were to assess safety and efficacy respectively. Fistula remission was complete closure of fistula openings with < 2 cm perianal collection on MRI, and fistula response was decrease in drainage by ≥ 50%. Change in perianal disease activity index, quality-of-life and Van Assche index on MRI over time was assessed using mixed-effect linear regression model.

**Results:**

Ten patients (male:8, mean age:27.4 ± 12.0years) were recruited. Self-resolving procedure-related adverse events occurred in three patients, with no follow-up adverse events. In intention to treat analysis at week 24, two patients (20%) achieved fistula remission and seven (70%) had fistula response. At week 52, two (20%) patients were in remission and seven (70%) maintained response. At 104 weeks, two (20%) patients maintained response and one (10%) was in remission. Statistically significant decrease in perianal disease activity index (*P* = 0.008), Van Assche Index (*P* = 0.008) and improvement in quality-of-life (*P* = 0.001) were observed over time.

**Conclusions:**

Allogeneic BMSCs are safe and effective for the treatment of perianal fistulizing CD with significant improvement in clinical severity and radiological healing.

**Trial registration:**

The study was prospectively registered on Clinical trials registry – India (CTRI), CTRI/2020/01/022743 on 14 January 2020, http://ctri.nic.in.

**Supplementary Information:**

The online version contains supplementary material available at 10.1186/s13287-024-03746-9.

## Background

Crohn’s disease (CD) is a chronic, multifactorial, immune mediated disease of the gastrointestinal (GI) tract characterized by stricturing and penetrating complications. Perianal fistula is one of the debilitating complications associated with considerable morbidity in patients with CD. Although, varying prevalence was reported in studies from different geographical regions across the world, approximately one-fifth of patients with CD are affected by perianal fistula at the time of diagnosis and one-third at 10 years following diagnosis [1]. Effective treatment options for treating perianal fistulizing CD are limited. Although various surgical techniques are available for the treatment of perianal CD, medical therapy remains cornerstone in the management to achieve and maintain remission [2]. However, despite the availability of multiple advanced medical therapies such as biologics and oral small molecules for the management of luminal CD, anti-TNF therapy was the only biological therapy that was systematically evaluated in phase 3 randomized controlled trials (RCTs) primarily designed for patients with perianal CD and remains preferred treatment of choice [3]. 

Mesenchymal stem cell (MSCs) therapy has shown to be safe and effective in patients with perianal CD in various studies with sustained long-term response and is a valuable addition to the existing therapeutic armamentarium for the management of perianal CD [4–6]. MSCs are multi-potent, spindle-like cells that possess the ability to self-renew as well as to differentiate into cartilage, bone and fat tissues in vitro [7]. MSCs exhibit unique immunomodulatory properties by suppressing T cell activation and proliferation, dendritic cell differentiation, maturation and function, B cell function, and natural killer cell proliferation [8]. MSCs can be allogenic or autologous and can be obtained from various tissues, such as adipose tissue and bone marrow [9, 10]. A large phase 3 RCT demonstrated statistically significant fistula response with human adipose tissue-derived MSCs (AMSCs) compared to placebo which led to approval of MSCs by European Medical Agency (EMA) as an orphan indication [6]. However, MSCs have been still undergoing evaluation in other regions of the world. Unlike AMSCs, efficacy of Bone marrow derived MSCs (BMSCs) has not been adequately investigated especially in Asia [11–14]. To the best of our knowledge, there are only four studies with small sample size available which evaluated safety and efficacy of BMSCs in adult patients with perianal fistulizing CD and none were conducted in Asian population [15–18]. Patients in Asian countries are genetically distinct with difference in gene polymorphisms which could potentially affect disease phenotype and response to therapy [19]. Majority of the studies evaluating efficacy of MSCs were conducted in European countries and these findings may not be directly applicable to individuals with perianal fistulizing CD in Asian populations. Therefore, it is crucial to assess safety of efficacy of MSCs which could potentially be useful in this population where there is limited availability of advanced therapies. Hence this phase I/II trial was undertaken to assess the safety and efficacy of local administration of human BMSCs in adult patients with perianal fistulizing CD.

## Methods

### Study design

An open label, single arm study was conducted for a total duration of 104 weeks (2 years), with the primary objective to assess the safety of local administration of adult human bone marrow derived, cultured, pooled, allogeneic mesenchymal stromal cells (BMSCs) in patients with perianal fistulizing CD. The secondary objective was to evaluate their efficacy by clinical and radiological assessments. The study was conducted in compliance with the protocol, the ethical principles that have their origin in the Declaration of Helsinki, the International Conference on Harmonization (ICH) consolidated Guideline E6 for Good Clinical Practice (GCP) (CPMP/ICH/135/95) and in accordance to “Guidelines for Stem Cell Research and Therapy” by Department of Biotechnology and Indian Council of Medical Research (ICMR), 2017, Schedule-Y and ICH-GCP and as per the recommendations of the Cellular Biology Based Therapeutic Drug Evaluation Committee (CBBTDE). The trial protocol was approved by institutional ethics committees and institutional stem cell committee (Ref No-IC-SCR/94/19) and is registered under clinical trials registry – India (CTRI No. CTRI/2020/01/022743). The confidentiality of all patients taking part in the study was preserved in accordance with GCP and local regulations. All patients provided written as well as audio-visual consent for participation in the study. The study recruitment began in February 2020 and completed in June 2022. Due to novel Coronavirus (SARS CoV-2) pandemic, for some follow up visits, patients could not visit hospital and hence the assessment was done telephonically for those visits. (Supplementary Table 1)

### Patient selection

Eligible patients were of either sex, aged between 18 and 65 years, with complex perianal fistulae associated with CD of at least 3 months duration, an active draining fistula with a maximum of 1 internal opening and a maximum of 2 external openings, that was refractory to medical (antibiotics, immunomodulators, or biologics) or surgical therapy. Patients were excluded if they had Crohn’s Disease Activity Index (CDAI) score more than or equal to 220 points, received steroids within 1 month prior to enrolment, treatment naïve fistulas, perianal abscess larger than 2 cm in diameter on magnetic resonance imaging (MRI) of the pelvis, presence of proctitis, anal canal stricture and fistulas other than perianal fistulas. Crohn’s disease was diagnosed as per the ECCO guidelines and were classified into various phenotypes using Montreal classification which includes age at onset, location and behaviour of the disase [20, 21] The investigations used for diagnosis included CT Enterography, ileocolonoscopy, and biopsy from abnormal mucosa. CT enterography was done for evaluation of small bowel in all patients. MR Pelvis was done for evaluation of the perianal fistula and presence of any perianal abscess or collection.

For the evaluation of perianal disease activity, PDAI score was used which includes variables like fistula discharge, pain/ restriction of activities, restriction of sexual activity, type of perianal disease, and degree of induration [22]. For the evaluation of luminal activity, CDAI score was used which includes the following variables: number of liquid stools, abdominal pain, general well-being, presence of extraintestinal complications, use of antidiarrheal drugs, presence of abdominal mass, body weight, and haematocrit [22]. 

For the evaluation of quality of life, a questionnaire comprising 5 questions and visual analogue scale was used and was rated from 0 to 100 with 0 being the worst control and 100 being the best control [23]. For the evaluation of radiological response, Van Assche index which included six MRI pelvis parameters: number of fistula tracts, fistula location and extension, T2 hyperintensity of the tract, presence or absence of collections and rectal wall involvement [24]. 

### Investigational Medical product (IMP)

STEMPEUCEL® is a suspension of 25 million ex vivo expanded, adult human bone marrow derived, cultured, pooled, allogeneic mesenchymal stromal cells (MSCs) formulated in CS5 medium and CZ vials. These MSCs were manufactured by Stempeutics Research Pvt. Ltd, Bengaluru, Karnataka, India, and registered as an Investigational medical product (IMP). The IMP was transported from the laboratory to the operating theatres of All India Institute of Medical Sciences (AIIMS), New Delhi, India in cryovial, in a temperature-controlled transport container containing a dry shipper that was stored in the transport container at -185 °C to -196 °C.

### Administration of mesenchymal stromal cells

**Fig. 1:**
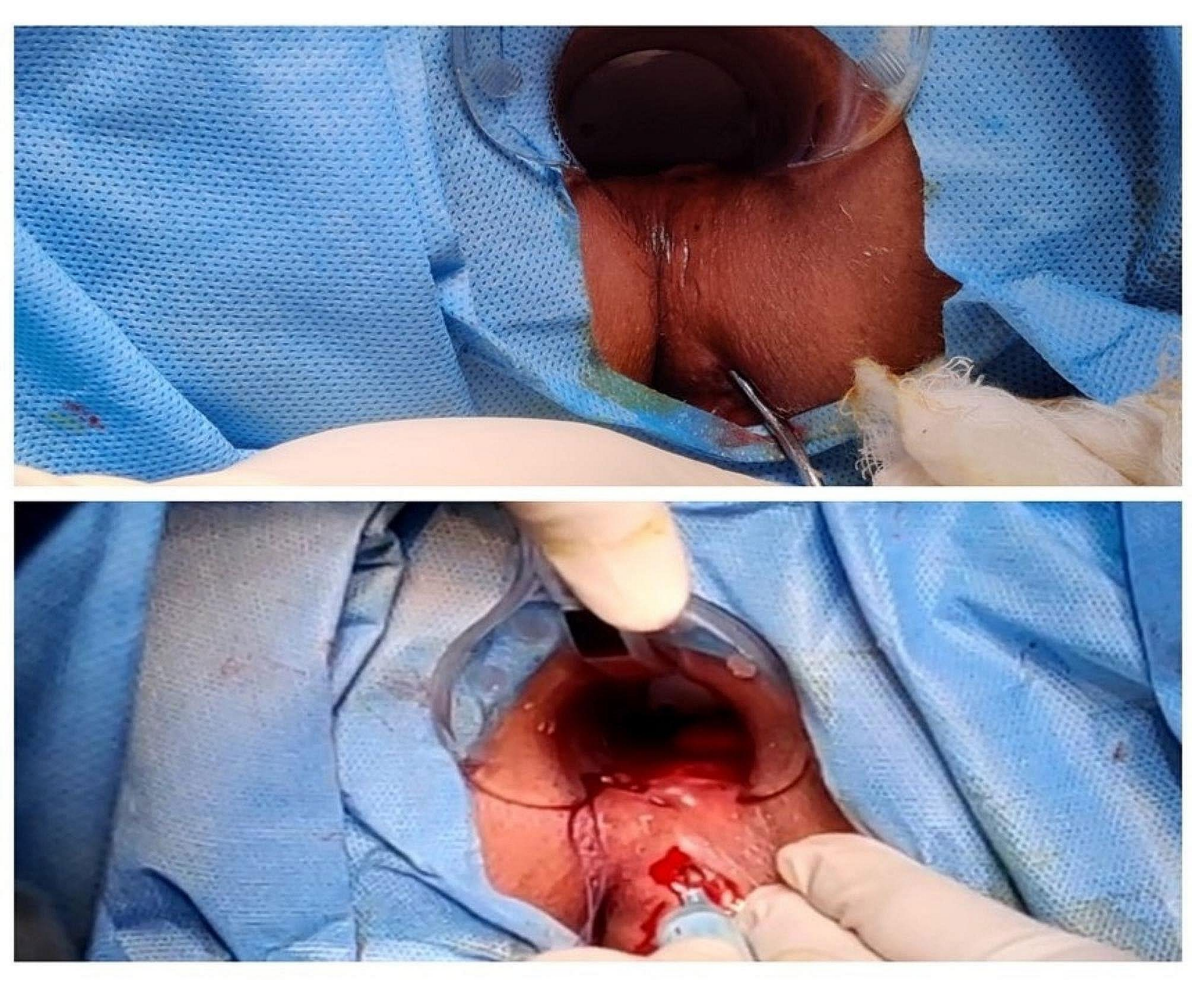
Administration of stem cells in perianal fistula

Before scheduling the administration, pre-medication with intravenous injection of 100 mg hydrocortisone and 45.5 mg of pheniramine maleate was administered, and administration of BMSCs was completed within 60 min of administration of first premedication. BMSC injections were administered locally through the perianal route under spinal anaesthesia. 75 million cells (15 mL cell suspension containing 5 × 10^6^ cells/mL) were provided through intralesional injection. Fistula tract was curetted, and internal opening was identified before administration of BMSCs. Internal opening was closed with absorbable sutures and 5 ml of cell suspension containing 25 million cells was injected at internal fistula opening. Remaining 10 ml cell suspension containing approximately 50 million cells was injected with 20 gauge long hypodermic needle along the walls of fistula tract so that it should produce a 2 mm bleb. Only one session of MSCs administration was done. Administration of BMSCs is depicted in Fig. 1. Patients were admitted in the hospital for 48 h after administration to monitor for acute local or systemic side effects.

### Baseline screening and follow up

The screening visit included clinical examination, vitals recording, blood tests, sigmoidoscopy or colonoscopy, and an MRI pelvis. BMSC injection was administered within 2 weeks of the screening visit. Crohn’s disease activity index (CDAI), Perianal Disease Activity Index (PDAI), quality of life (assessed as visual analogue scale), and evaluation of adverse events were assessed for all participants at baseline as well as at 2, 6, 12, 18, 24, 52 and 104 weeks. MRI Pelvis and sigmoidoscopy or colonoscopy were repeated at 24 weeks and 104 weeks during follow up. Details of follow up visit is provided in Supplementary Table 1.

### Outcome measures

An adverse event was defined as any untoward medical occurrence in a patient administered IMP and which did not necessarily have a causal relationship with treatment at weeks 0, 12, 24, 52, 104 and has been graded as per Common terminology criteria for adverse events (CTCAE) V5. Fistula remission was defined as complete closure of all external openings and no collections larger than 2 cm on pelvic MRI at weeks 24 and 104. Fistula response was defined as closure of more than 50% of all openings or a decrease in fistula discharge by ≥ 50%. Change in PDAI, and quality-of-life was assessed at weeks 24, 52 and 104. Quality-of-life was assessed as a visual analogue scale (VAS) ranging from 0 to 100 (worst to best) [23]. Change in Van Assche index (VAI) was assessed at week 24 and 104 weeks [24]. 

### Statistical analysis

Statistical analysis was performed using standard methods. Continuous variables that were normally distributed were expressed as mean ± standard deviation(SD), otherwise expressed as median (range). Categorical data were presented as proportions. Changes in the four outcome measures (PDAI, QOL score, CDAI and Van Assche Index score) over time was assessed using mixed-effect linear regression model. Wilcoxon sign rank test was used to compare median measurements of these four outcomes for each pair of follow-up duration. A p-value < 0.05 was considered statistically significant. Statistical analysis was performed using Stata v14 (StataCorp, Texas, USA).

### Role of the funding source

This study was supported and funded by Stempeutics Research Pvt. Ltd, Bengaluru, Karnataka. The funder of the study had no role in the data collection, data analysis, data interpretation, or writing of the report.

## Results

### Baseline characteristics

**Fig. 2:**
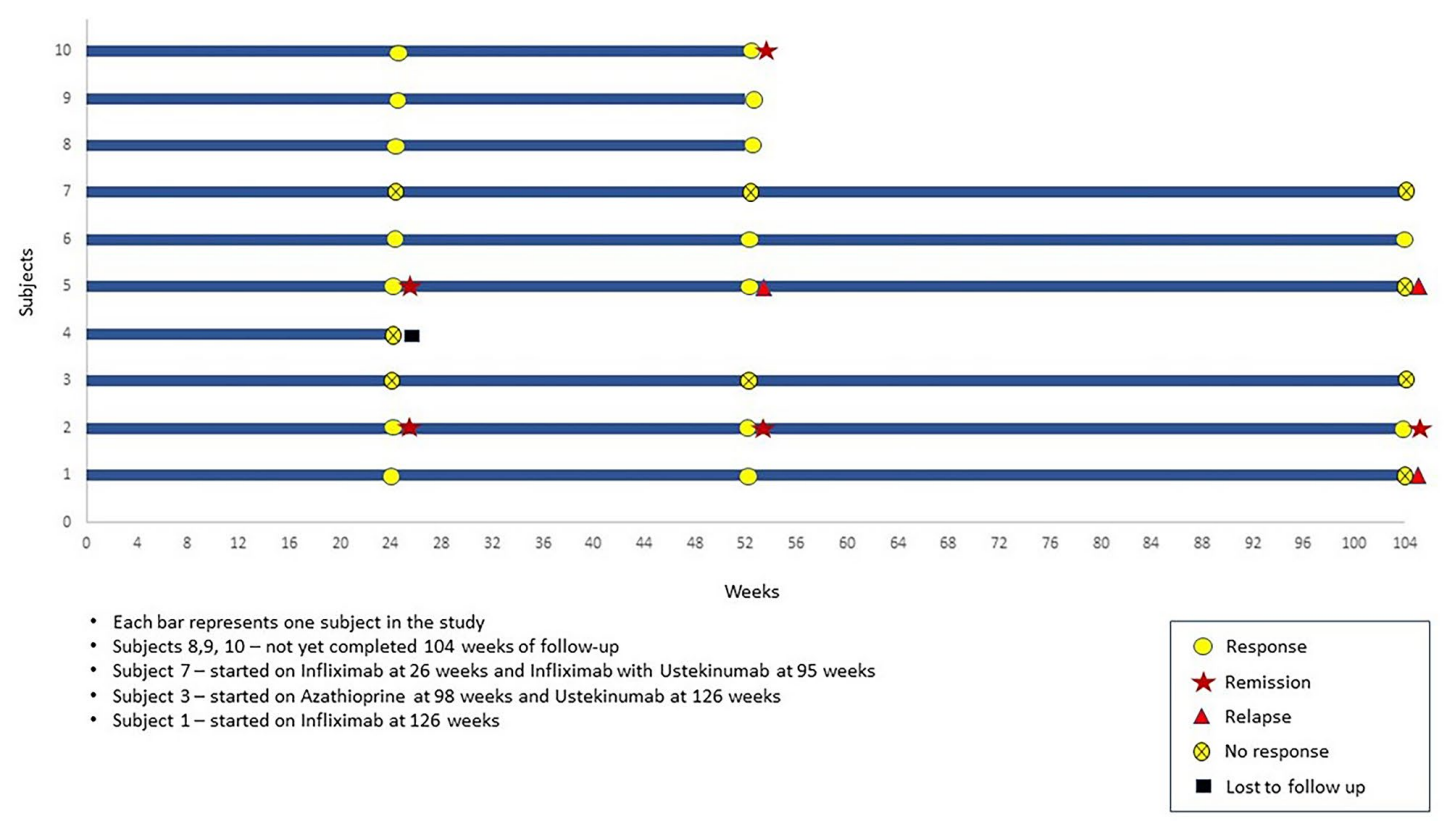
Swimmer’s plot: Outcome measures over time

Ten CD patients with actively draining perianal fistula (eight males, mean age − 27.4 ± 12.0 years) were recruited after satisfying eligibility criteria. Median disease duration was 7.5 (IQR: 2.5–21.0) years. All patients failed medical therapy and six patients failed both medical and surgical therapies prior to recruitment. None of the patients were on anti-TNF therapy at the time of recruitment. All 10 patients received multiple courses of antibiotics for variable duration ranging from 1 month to 10 years without any response in terms of fistula healing. Commonly used antibiotics were Ciprofloxacin, Ofloxacin, Metronidazole, and Satranidazole. Seven out of 10 patients also had received immunomodulators in the form of Azathioprine for a duration ranging from 6 months to 6 years. Eight patients also have been on multiple courses of steroids including budesonide and prednisolone. Biological therapy with Infliximab was received by 5 patients and 1 patient received both infliximab and adalimumab. Details of previous therapy can be found in the Table 1. Majority had colonic involvement (5/10) and none of the patients had non-perianal fistulae. One patient had exclusive perianal involvement without significant bowel involvement. All patients who were recruited had received multiple courses of antibiotics in the past. Baseline characteristics of the patients are shown in Table 1. Clinical course of patients during the study is summarized in a Swimmer’s plot in Fig. 2.

**Table 1:** Baseline Characteristics

Characteristics	*N* = 10
Female gender n (%)	2 (20%)
Age (years)	27.4 ± 12.0
Montreal Classification	
L1 L2 L3 Only perianal disease B1 B2 B3	3 (30%) 5 (50%) 1 (10%) 1 (10%) 9 (90%) 1 (10%) 0 (0%)
Previous Surgery	
Seton placement VAAFT Diversion Ileostomy Fistulotomy	2 (20%) 2 (20%) 1 (10%) 1 (10%)
Previous Medical Treatment	
Antibiotics Steroids Immunomodulators Biologicals	10 (100%) 8 (80%) 7 (70%) 6 (60%)
Median duration (IQR), years	7.5 (2.5–21.0)
No of external openings	
1 2	8 (80%) 2 (20%)
Median PDAI score (median [IQR])	9 (7 - 9)
Median CDAI score (median [IQR])	66 (50–102)
Median IBD QOL (VAS) score (median [IQR])	30 (20–30)

On clinical assessment at baseline, eight out of ten patients had one external opening and two patients had two external openings. The median PDAI score at baseline was 9 (IQR:7–9), the median IBD-QOL (VAS) score was 30 (IQR: 20–30) and the median CDAI score was 66 (IQR: 50–102). (Table 1)

On radiological assessment at baseline, four patients had inter-sphincteric, one patient had extra-sphincteric, two patients had trans-sphincteric, and three patients had supra-sphincteric location of fistula. Seven patients had presence of collections which were less than 2 cm. Seven patients had moderate and three had mild T2 hyperintensity on MRI. Three patients had rectal wall thickening despite no evidence of active proctitis on sigmoidoscopy. Mean Van Assche Index was 15.1 ± 5.4. (Table 2)

**Table 2:** MRI characteristics over time

Characteristics	Baseline	24-52 weeks *N* = 8*	104 weeks *N* = 6#	
No of fistula				
• single, unbranched • single, branched • multiple	2 (20%) 6 (60%) 2 (20%)	3 (39%) 5 (63%) 0 (0%)	4 (66%) 1 (17%) 1 (17%)	
Location				
• extra- or intersphincteric • transsphincteric • suprasphincteric	5 (50%) 2 (20%) 3 (30%)	6 (75%) 0 (0%) 2 (25%)	4 (66%) 2 (34%) 0 (0%)	
Extension				
• infralevatoric • supralevatoric	7 (70%) 3 (30%)	6 (75%) 2 (25%)	6 (100%) 0 (0%)	
T2 hyperintensity				
• absent • mild • pronounced	0 (0%) 3 (30%) 7 (70%)	1 (13%) 2 (25%) 5 (62%)	1 (16%) 5 (84%) 0 (0%)	
Collections (> 3 mm)				
• absent • present	3 (30%) 7 (70%)	1 (13%) 7 (87%)	3 (50%) 3 (50%)	
Rectal wall involvement				
• normal • thickened	7 (70%) 3 (30%)	6 (75%) 2 (25%)	5 (84%) 1 (16%)	
Van Assche Score (Mean ± SD)	15.3 ± 5.4	14.4 ± 5.1	9.5 ± 4.0	0.008

*1 patient lost to follow up at 24 weeks, 1 patient could not get MRI done due to COVID Pandemic

#1 patient lost to follow up at 24 weeks, 3 patients yet to complete 104 weeks follow up

### Safety of BMSCs

Three out of the ten patients had periprocedural adverse events in the form of post spinal headache in two patients which was considered unrelated to the BMSCs injection. One patient had perianal ecchymoses and urinary retention following procedure which resolved without requiring any medical of surgical intervention. Seven patients had perianal pain which required analgesics. All events were mild in severity. One patient with no response to stem cell therapy, on dual biological therapy (Infliximab and Ustekinumab) developed left iliac fossa abscess at 100 weeks and was treated with antibiotics and drainage of abscess. Adverse events during procedure and follow up are shown in Tables 3 and 4.

**Table 3:** Safety outcome

		Grade of adverse events		
		Mild	Moderate	Severe
	Subjects at risk			
0 weeks	10	7	0	0
24 weeks	9^#^	0	1*	0
52 weeks	9^#^	0	0	0
104 weeks	6^$^	0	0	1*

* - Disease related

^#^ − 1 patient lost to follow up at 24 weeks

^$^ − 3 patients to complete their follow up of 104 weeks, 1 patient lost to follow up at 104 weeks

**Table 4:** Adverse events

Immediate adverse events:			
	Number	Severity*	Outcome
Procedure Related			
Perianal pain	7	Grade 1	Required analgesics
Post spinal headache	2	Grade 1	Resolved without intervention
Urinary retention	1	Grade 1	Resolved without intervention
Drug Related			
Perianal ecchymoses	1	Grade 1	Resolved without intervention
Delayed adverse events during follow-up:			
Procedure Related	None		
Drug Related	None		
Disease Related			
Increased luminal activity (Increased CDAI score)	1	Grade 2	Response on biological
Left Illiac fossa abscess	1	Grade 3	Resolved with drainage and antibiotics

*Common Terminology Criteria for Adverse Events (CTCAE) v5

### Efficacy of BMSCs

#### Clinical assessment

A total of ten patients were recruited, one of whom was lost to follow up after week 24. Remaining nine patients had completed 52 weeks of follow up and five patients had completed week 104 follow up.

At week 24, two (20%) patients achieved fistula remission and seven (70%) achieved fistula response and none of these patients received any concomitant biological therapy or surgical drainage. Among the three patients who did not achieve fistula response at week 24 were managed with different therapeutic strategies. The first patient was started on biological with Infliximab at 26 weeks and subsequently upgraded to a combination of Infliximab and Ustekinumab at 95 weeks. The second patient was started on Azathioprine at 98 weeks and Ustekinumab at 126 weeks. The third patient underwent seton placement and fistulectomy at 24 weeks. Among the seven patients who achieved response at 24 weeks, 1 patient was started on Azathioprine at 52 weeks with a course of antibiotics. One patient required the addition of a course of antibiotics during follow-up. The rest of 5 patients did not receive any concomitant therapy (antibiotics, immunomodulators, steroids or biologicals) during follow up. On per protocol analysis at week 24, two patients (20%) achieved fistula remission and seven (70%) fistula response. At week 52, one patient who had remission at 24 weeks relapsed, one patient maintained remission, one more patient achieved remission, hence two out of nine (22%) patient were in remission and seven out of nine (78%) maintained response. At 104 weeks, two out of six (33%) patients maintained response and one (17%) patient maintained remission. On intention to treat analysis at week 24, two patients (20%) achieved fistula remission and seven (70%) fistula response. At week 52, two out of ten (20%) patient were in remission and seven out of ten (70%) maintained response. At 104 weeks, two out of ten (20%) patients maintained response and one (10%) patient was in remission. Outcome measures are shown in Tables 5 and 6; Fig. 2 for the study population.

**Table 5:** Outcome measures: Remission, Response and Relapse

		Intention to treat analysis^$^	Per Protocol analysis^#^
24 weeks	Response	7 (70%)	7 (70%)
	Remission	2 (20%)	2 (20%)
52 weeks	Response	7 (70%)	7 (78%)
	Remission*	2 (20%)	2 (22%)
104 weeks	Response	2 (20%)	2 (33%)
	Remission	1 (10%)	1 (17%)

*Remission was complete closure of all external fistulas, no MRI done at 52 weeks

^$^*N* = 10 was taken at all time points for intention to treat analysis

^#^*N* = 10 at 24 weeks, *N* = 9 at 52 weeks, *N* = 6 at 104 weeks

**Table 6:** Time to response, remission and relapse

Time to response (*n* = 7)	Number of paticipants
24 weeks 52 weeks 104 weeks	7 0 0
Time to Remission (*n* = 3)	
24 weeks 52 weeks* 104 weeks	2 1 0
Time to relapse	Time in weeks
With prior remission (*n* = 1) With prior response (*n* = 1)	52 weeks 104 weeks

*Remission was complete closure of all external fistulas, no MRI done at 52 weeks

#### Patient reported outcome measures

**Fig. 3:**
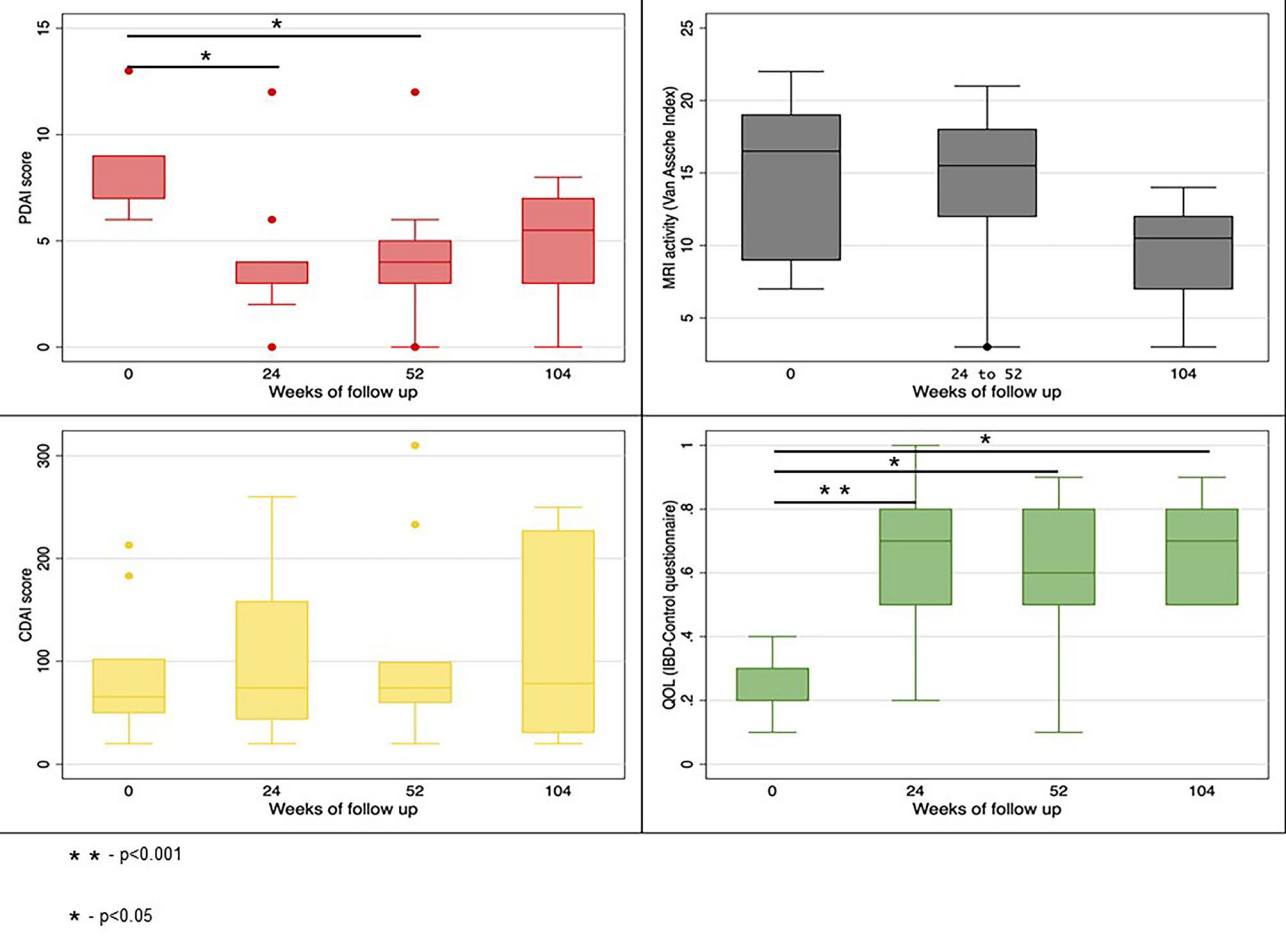
Change in various scores from baseline to weeks 104

**Fig. 4:**
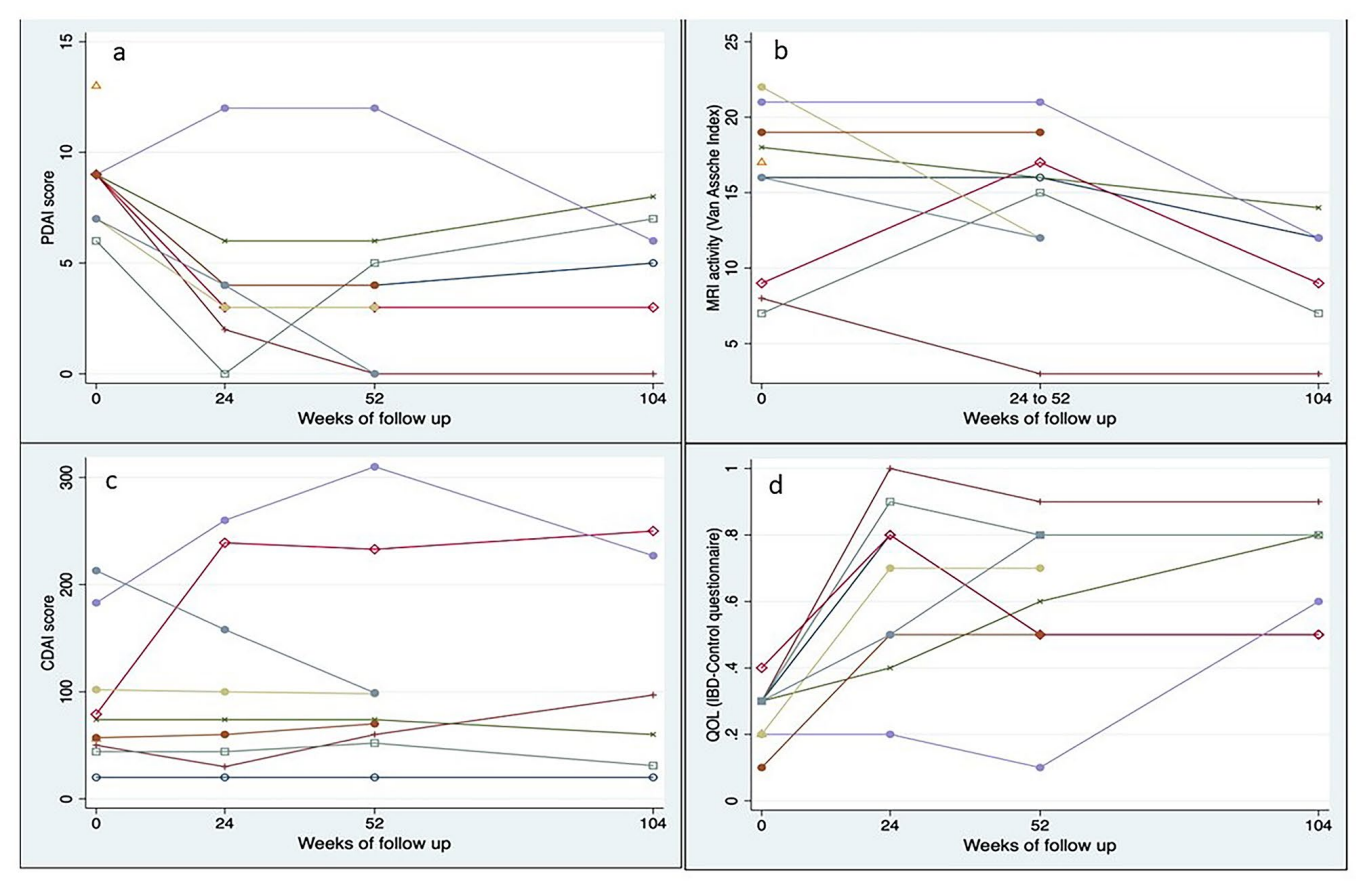
Evolution per patient of the PDAI score, Van Assche Index, CDAI score, QOL score

Median PDAI (IQR) scores at baseline, 24 weeks, 52 weeks, and 104 weeks were 9 (7.0–9.0), 4 (3.0–4.0), 4 (3.0–5.0) and 5.5 (3.0–7.0) respectively (*P* = 0.008). Median IBD QOL (VAS) (IQR) scores at baseline, 24 weeks, 52 weeks, and 104 weeks were 30 (20–30),70 (50–80), 60 (50–80) and 70 (50–80) respectively (*P* = 0.001). Median CDAI (IQR) scores at baseline, 24 weeks, 52 weeks, and 104 weeks were 66 (50–102), 74 (44–158), 74 (60–99), and 79 (31–227) (*P* = 0.251). (Table 7; Fig. 3) One patient had worsening of luminal activity, indicated by an increasing CDAI score, and requiring use of Infliximab at week 52. Evolution per patient of PDAI, IBD-QOL (VAS) score, and CDAI over time is depicted in Fig. 4.

**Table 7:** Changes in PDAI, IBD QOL and CDAI scores over time

	Baseline (*n* = 10)	24 weeks* (*n* = 9)	52 weeks (*n* = 9)	104 weeks (*n* = 6)	*P* value
PDAI score Median (IQR)	9.0 (7.0–9.0)	4.0 (3.0–4.0)	4 (3.0–5.0)	5.5 (3.0–7.0)	0.008
IBD QOL Median (IQR)	30 (20–30)	70 (50–80)	60 (50–80)	70 (50–80)	0.001
CDAI Median (IQR)	66 (50–102)	74 (44–158)	74 (60–99)	79 (31–227)	0.251

*1 patient lost to follow up at 24 weeks

#### Radiological assessment

**Fig. 5:**
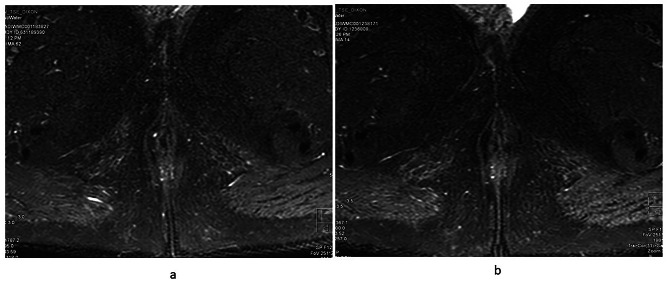
MRI image of a patient in remission (**a**) At baseline - a linear fistulous tract is seen which is hyperintense on T2W sequence, (**b**) at 104 weeks – the fistulous tract becomes hypointense on T2W suggestive of fibrosis

The mean VAI score at baseline was 15.3 ± 5.4 which showed statistically significant decline over time with VAI score of 14.4 ± 5.1 at 24–52 weeks, and 9.5 ± 4.0 at 104 weeks (*P* = 0.008). Seven out of ten patients had pronounced T2 hyperintensity of the fistulous tract at baseline, five out of eight had pronounced T2 hyperintensity at 24–52 weeks and none of the patients had T2 hyperintense fistulous tract at 104 weeks (Table 2; Fig. 5).

## Discussion

Efficacy and safety of adipose derived MSCs in perianal fistulizing CD has been documented in various studies, and they have shown promise in the management of this complex condition [15–18]. However, there is lack of robust data on efficacy of BMSCs. In the present phase I/II study we have demonstrated that BMSCs are safe in patients with CD having complex perianal fistulae after failure of conventional medical and surgical therapies. Apart from safety, BMSCs were also effective in achieving fistula remission and response, and corresponding improvement in quality of life.

Local administration of MSCs is considered safe without any significant increased risk of adverse effects compared to placebo across clinical trials. In a large RCT comparing local administration of AMSCs (ADMIRE-CD), 17% of patients receiving MSCs developed treatment related adverse events compared to 29% in placebo arm at week 24 [6]. The most common adverse event was perianal abscess, and it was considered to be unrelated to MSCs but instead due to manipulation of perianal tissues. In the long-term follow up of same study, seven out of 40 patients had treatment emergent adverse events through 104 weeks [4]. Studies on BMSCs also demonstrated no increase in adverse events. In a placebo-controlled trial assessing local administration of allogenic BMSCs with three different doses of stem cells compared with placebo, no serious adverse events were reported except for one perianal abscess event in each group and one patient with positive family history of colorectal cancer receiving MSCs developed caecal carcinoma which was considered unlikely to be a result of stem cell therapy [15]. In our study, in consistence with results of previously published studies, we did not observe serious adverse events in patients receiving BMSCs. Similarly, a recently published study performed in paediatric patients with perianal fistulizing CD in seven participants did not demonstrate any serious adverse events [17]. 

As far as efficacy is concerned, local administration of MSCs demonstrated statistically significant clinical as well as radiological improvement across various studies. In ADMIRE-CD trial, patients randomized to AMSCs arm achieved combined clinical and radiological remission in 50% of patients when compared to 34% in placebo arm at 24 weeks. High response rates in placebo arm could be because of surgical treatment received in placebo arm along with placebo [6]. Long term follow up also demonstrated sustained remission [4]. Studies on BMSCs showed varying fistula healing ranging from 20 to 83% [15–18]. In our study 70% of patients experienced fistula response and 20% achieved remission at 24 weeks.

Although, all types of MSCs are presumed to have similar properties, several studies have demonstrated considerable differences in immunomodulatory properties [25, 26]. Comparative studies on different types of MSCs demonstrated notable differences at molecular level as well as in clinical efficacy between AMSCs and BMSCs [27–30]. This suggests that there could be potential therapeutic differences between AMSCs and BMSCs in the management of perianal fistulizing CD which needs to be further explored. Genetic and phenotypic differences in inflammatory bowel disease between Western and Asian population may also influence the efficacy of MSCs [31–33]. Therefore, our study is a valuable addition to the existing limited literature.

### Limitations of the study

First, our study is a single center study with small sample size and majority being males, limiting generalizability of results. Moreover, the genetic background of patients was similar. However, prospective long-term follow-up for two years demonstrated safety of MSCs. Secondly, there was no control arm, therefore comparative efficacy with standard of care was not possible. We did not include patients with more than two external openings, therefore, results of our study many not be applicable to patients with multiple fistula tracts and external openings. All patients did not undergo surgical drainage/seton placement prior to stem cell administration, hence the response when combined with drainage could not be assessed. Furthermore we did not explore the mechanistic property of mesenchymal stromal cells which would involve the measurements of inflammatory cytokines in the serum, rectal tissue and perianal fistula scraping. Lastly, a single dose of MSCs was administered as was the practice in previous clinical trials of MSCs [6, 18]; a repeat injection in those who achieved partial response or inadequate response may be required to achieve optimal response. In a recent paediatric study repeat injection of BMSCs after 3 months, if there was no response, led to complete clinical and radiological healing in 83% of patients [17]. 

The mechanistic aspects of BMDSc like inflammatory cytokines, change in microbiota were not assessed, which would have made our conclusion more strong.

## Conclusion

To conclude, our study findings demonstrated that allogeneic BMSCs are safe and effective in patients with perianal fistulizing CD refractory to conventional therapy.

### Electronic supplementary material

Below is the link to the electronic supplementary material.

Supplementary Material 1

## Data Availability

The data can be made available and shared upon reasonable request from the corresponding author depending on the nature of the request and its intended use.

## Abbreviations

AIIMS: All India Institute of Medical Sciences
AMSCs: adipose tissue-derived mesenchymal stem cells
BMSCs: bone marrow-derived mesenchymal stem cells
CBBTDE: Cellular Biology Based Therapeutic Drug Evaluation Committee
CD: Crohn’s Disease
CDAI: Crohn’s Disease Activity Index
COVID: Coronavirus disease
CTRI: Clinical trials registry – India
EMA: European Medical Agency
GCP: Good Clinical Practice
GI: Gastrointestinal
IBD-QoL: inflammatory bowel disease quality of life
ICH: International Conference on Harmonization
ICMR: Indian Council of Medical Research
IMP: Investigational Medical Product
MRI: magnetic resonance imaging
MSCs: mesenchymal stem cells
MSCT: mesenchymal stem cell therapy
PDAI: Perianal disease activity index
RCTs: Randomised control trials
VAI: Van Assche index
