# Supplementary material for: A phase I/II clinical trial of ex-vivo expanded human bone marrow derived allogeneic mesenchymal stromal cells in adult patients with perianal fistulizing Crohn’s Disease
Source: Stem Cell Res Ther. 2024 May 14;15:140. doi: 10.1186/s13287-024-03746-9 (PMC11094973; doi:10.1186/s13287-024-03746-9)
Supplement: Supplementary file 1 — Supplementary Material 1 [file 13287_2024_3746_MOESM1_ESM.docx]

| Patient | Visit 1  Screening | Visit 2  Administration of stem cell  (Week 0) | Visit 3  (Week 2) | Visit 4  (Week 6) | Visit 5  (Week 12) | Visit 6  (Week 18) | Visit 7  (Week 24) | Visit 8  (Week 52) | Visit 9  (Week 104) |
| --- | --- | --- | --- | --- | --- | --- | --- | --- | --- |
| 1. | ✔ | ✔ | ✔ | X | X | X | ✔ | ✔ | ✔ |
| 2. | ✔ | ✔ | X | X | X | X | ✔ | ✔ | ✔ |
| 3. | ✔ | ✔ | ✔ | X | X | X | ✔ | ✔ | ✔ |
| 4. | ✔ | ✔ | ✔ | ✔ | X | X | - | - | - |
| 5. | ✔ | ✔ | ✔ | ✔ | X | X | ✔ | ✔ | ✔ |
| 6. | ✔ | ✔ | ✔ | X | X | X | ✔ | ✔ | ✔ |
| 7. | ✔ | ✔ | ✔ | ✔ | X | ✔ | ✔ | ✔ | ✔ |
| 8. | ✔ | ✔ | X | X | X | X | ✔ | ✔ | * |
| 9. | ✔ | ✔ | X | X | X | X | ✔ | ✔ | * |
| 10. | ✔ | ✔ | X | X | ✔ | ✔ | ✔ | ✔ | * |

Supplementary table 1: Follow ups of the patient

“X”- Patient could not follow up due to COVID pandemic

“✔”- Patient came at the specified visit

“-“ - Lost to follow up

“*”- Follow up to be done

PDAI score, CDAI score and IBD QOL score was calculated over telephonic call when patient could not visit hospital
